# Invariance (?) of Mutational Parameters for Relative Fitness Over 400 Generations of Mutation Accumulation in *Caenorhabditis elegans*

**DOI:** 10.1534/g3.112.003947

**Published:** 2012-12-01

**Authors:** Chikako Matsuba, Suzanna Lewis, Dejerianne G. Ostrow, Matthew P. Salomon, Laurence Sylvestre, Brandon Tabman, Judit Ungvari-Martin, Charles F. Baer

**Affiliations:** * Department of Biology, University of Florida, Gainesville, Florida 32611-8525; † UF Genetics Institute, Gainesville, Florida 32610-3610

**Keywords:** Bateman-Mukai method, fitness-dependent mutation, genetic load, mutational variance

## Abstract

Evidence is accumulating that individuals in poor physiologic condition may accumulate mutational damage faster than individuals in good condition. If poor condition results from pre-existing deleterious mutations, the result is “fitness-dependent mutation rate,” which has interesting theoretical implications. Here we report a study in which 10 mutation accumulation (MA) lines of the nematode *Caenorhabditis elegans* that had previously accumulated mutations for 250 generations under relaxed selection were expanded into sets of “second-order” MA lines and allowed to accumulate mutations for an additional 150 generations. The 10 lines were chosen on the basis of the relative change in fitness over the first 250 generations of MA, five high-fitness lines and five low-fitness lines. On average, the mutational properties (per-generation change in mean relative fitness, mutational variance, and Bateman-Mukai estimates of genomic mutation rate and average mutational effect) of the high-fitness and low-fitness did not differ significantly, and averaged over all lines, the point estimates were extremely close to those of the first-order MA experiment after 200 generations of MA. However, several nonsignificant trends indicate that low-fitness lines may in fact be more likely to suffer mutational damage than high-fitness lines.

It is now widely appreciated that mutational properties—rate, molecular spectrum, and phenotypic effects—vary at all taxonomic levels, including among individuals within populations (*e.g*., Drake *et al.* 1998; Lynch 2010). The proximate (mechanistic) and ultimate (evolutionary) causes for that variation in most cases are not well understood. One possibility that has gained appreciation in recent years is that mutation may be condition-dependent, such that individuals in poor physiologic condition experience a greater probability of mutation than individuals in good condition (Goho and Bell 2000; Agrawal and Wang 2008; Baer 2008a; Sharp and Agrawal 2012). If physiologic condition and fitness covary, it follows that individuals with low fitness may have greater rates of mutation than individuals with high fitness. If fitness-dependent mutation rate (FDMR) turns out to be a general phenomenon, there are important theoretical implications for several aspects of evolutionary biology (Agrawal 2002; Shaw and Baer 2011).

A related, potentially confounding phenomenon concerns the phenotypic effects of mutations. If the effects of a given mutation are more severe in an individual in poor condition—for which the evidence is at least suggestive—it follows that deleterious mutations should manifest negative (synergistic) epistasis, in which the deleterious effects of a mutation increase with the number of deleterious alleles present in the genome.

FDMR and negative epistasis obviously are not mutually exclusive. However, if at least one of the two possibilities applies, the expectation is that fitness will decrease progressively faster with the accumulation of deleterious mutations. If fitness does not decrease progressively faster with mutation accumulation (MA), the likely explanation is that neither FDMR nor negative epistasis applies; the unlikely explanation is that FDMR and epistasis interact in a peculiar way such that the decline in fitness remains linear. Unambiguously attributing a nonlinear rate of change of fitness to either of the two potential causes requires both characterization of the genome-wide mutation rate and an estimate of mutational effects on fitness.

Here we report the results of an experiment designed to establish the relationship between starting mutation load and the subsequent change in fitness with MA. We measured fitness of a set of MA lines of the N2 strain of the nematode *Caenorhabditis elegans* that had undergone 250 generations of MA (Baer *et al.* 2006). From this initial set of 67 “first-order MA” (1° MA) lines, we chose five lines with consistently high absolute fitness (measured as lifetime reproductive output, *W*) and five lines with consistently low fitness and initiated a “second-order MA” experiment (2° MA), in which each of the 10 starting 1° MA lines was the founder of its own set of 48 2° A lines. We then measured the change in the mean and genetic variance of fitness in a subset of 2° MA lines after an additional 150 generations of MA and used these measures to estimate the genomic mutation rate (*U_MIN_*) and the average effect of a mutation (E[*a*]*_MAX_*) using the Bateman-Mukai method (Bateman 1959; Mukai 1964).

To our knowledge, there has been only one other second-order MA experiment in a multicellular eukaryote. Ávila *et al.* (2006) conducted such an experiment with a single high-fitness 1° MA line of *Drosophila melanogaster*. They found that the rate of decrease of fitness increased in the 2° MA lines. The authors interpreted the accelerating decline in fitness as the result of an increase in genomic mutation rate for fitness (*U*), but the decline is consistent with negative epistasis, and some authors have interpreted their results as providing evidence that epistasis is negative (*e.g.*, Dickinson 2008).

## Materials and Methods

### MA protocol

**Figure 1:**
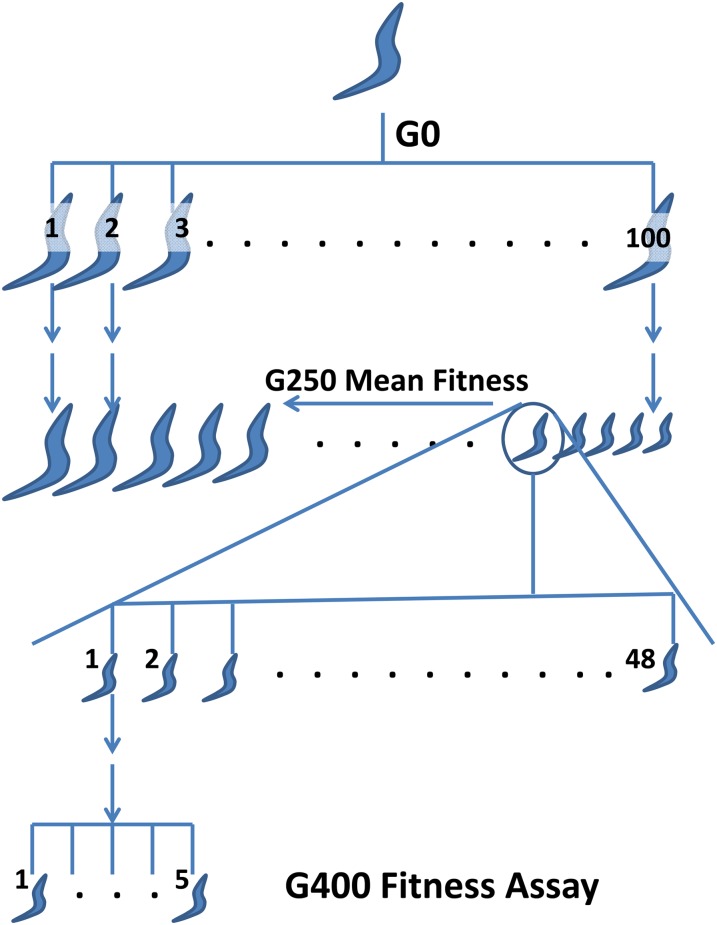
Schematic depiction of the MA protocol. Lines designated 1, 2, … 100 branching from the worm labeled G0 represent 1° MA lines. G250 represents 250 generations of MA, size of the worm represents fitness, ordered by decreasing fitness. The circled worm represents one low-fitness 1° MA line; lines designated 1,2, … 48 branching from the circled worm represent 2° MA sublines. The five worms designated 1…5 at generation 400 represent the five replicates of that line in the fitness assay.

An overview of the experimental design is depicted in Figure 1. Details of the 1° MA protocol and fitness assays are reported in Baer *et al.* (2005). In summary, MA was initiated in the spring of 2003 with 100 replicate lines derived from a single highly inbred individual N2 strain hermaphrodite. Fitness was assayed after 100 and 200 generations of MA (Gmax) at 20° and at 25° at Gmax = 220 (Baer *et al.* 2006). MA was carried out until Gmax = 250 and all surviving lines were cryopreserved using standard methods (Wood 1988). On the basis of the results of these fitness assays, we initially selected 21 lines from the tails of the fitness distribution (10 high, 11 low) and heuristically characterized their fitness as “putative high” or “putative low.” Of those lines, we chose seven putatively high and seven putatively low fitness lines for quantitative measurement of fitness. Fitness of these lines was assessed by the method described in Baer *et al.* (2005) except we used 10 replicates per line rather than five, and we only carried out one generation of single-worm descent before assaying fitness; thus, parental and grandparental effects potentially contribute to differences in fitness. Of these lines, we chose the five low lines with the lowest fitness and the five high lines with the highest fitness from which to initiate sets of 2° MA lines.

In what follows, we refer to the 10 progenitor stocks of the second-order MA experiment as “first-order MA lines” (1° MA) and we refer to the set of second-order MA lines (2° MA) derived from an individual 1° MA progenitor as “sublines.” In the spring of 2008, populations were begun from a single immature hermaphrodite from each of the 10 cryopreserved 1° MA progenitors, expanded, and 48 2° MA sublines established from each progenitor.

The 2° MA protocol was essentially identical to the original 1° MA protocol described in Baer *et al.* (2005). Worms were kept at 20° on NGM agar plates (60 mm diameter) seeded with 100 μL of overnight culture of the OP50 strain of *Escherichia coli*. Sublines were initially propagated by transferring a single immature (L4 stage) hermaphrodite to new plate at 4-day intervals. It shortly became apparent that four of the five 1° low-fitness progenitors had evolved slower generation times, so those lines were changed to a 5-day generation time a few months after the initiation of 2° MA. At every generation the previous two generations of each subline were kept as backup; at any generation if the leading generation worm did not reproduce, the plate was reinitiated with an immature individual from the previous generation backup plate. “Going to backup” does not affect the total number of generations of MA (*i.e.*, Gmax); it does affect the effective population size (*N_e_*) and thus the parameter of effective neutrality (4*N_e_s* < 1; see discussion in Baer *et al.* (2010). Mean effectively neutral selection coefficients (*s*_n_) were very slightly smaller in the low-fitness lines $\left({\overset{–}{s}}_{n}\;=\;\;0.22\right)$ than in the high-fitness lines $\left({\overset{–}{s}}_{n}\;=\;\;0.24\right)$; see supporting information, Table S3. Lines with 4-day generation times reached Gmax = 150 (G400) and were cryopreserved in January 2010; 5-day generation lines reached Gmax = 150 (G400) and were cryopreserved in June 2010.

### Fitness assay

The fitness assay protocol is described in detail in Baer *et al.* (2005). Each 1° line was initially represented by up to 24 2° sublines and 10 replicate “pseudolines” of each 1° progenitor; “pseudolines” are derived from immature individuals recovered from thawed plates prior to reproduction and are in principle genetically homogeneous. We included 15 pseudolines of the G0 N2 common-ancestor as an additional control. Cryopreserved stocks (2° sublines, 1° progenitors, and the G0 N2 common ancestor) were simultaneously thawed onto seeded NGM plates. The among-pseudoline component of variance represents variation (for whatever reason) among cryopreserved individuals recovered after thawing. All 2° sublines and 1° pseudolines were replicated 5X from immature individuals taken directly from the thawed plate and propagated for three generations by single-individual descent (P1-3) at 4- or 5-day intervals, respectively. Plates were assigned random numbers, and after the first generation (P1) were identified only by the random number and handled in random numerical order. A single newly-hatched (L1) offspring of the P3 parent was picked on new plate (called R1, R represents “reproduction”). After 2 days, the grown adult worm was transferred to new plate (R2) and transferred again the following day (R3). R-plates were incubated at 20° for 24 hr to allow eggs to hatch and then stored at 4°. Subsequent to completion of the assay, stored plates were stained with 0.075% toluidine blue and the worms on the plates were counted under a dissecting microscope at ×20 magnification.

One low-fitness line (line 579) unexpectedly increased in fitness over the 150 generations of 2° MA (Table 1), an unprecedented result in our MA experiments with Caenorhabditis. We reassayed line 579 with a larger number of control pseudolines. The point estimate of the per-generation change in mean fitness in the re-assay was slightly negative but not significantly different from the original assay, so data from both assays are included in subsequent analyses unless noted otherwise.

**Table 1:** Summary statistics of mean fitness

1° Line	1° Fitness	n_sublines (1°, 2° MA)	*W_0_*	*W_MA_*	*w*_0_	*w_MA_*	Δ*M_w_* (×10^3^)
504	Low	10, 22	58.3 (6.8)	37.4 (4.6)	1 (0.01)	0.57 (0.10)	−2.84 (−4.01, −1.24)
508	Low	10, 21	39.5 (4.7)	23.1 (2.7)	1 (0.00)	0.58 (0.09)	−2.80 (−3.85, −1.60)
547	Low	10, 22	47.9 (7.3)	37.5 (4.5)	1 (0.01)	0.67 (0.15)	−2.22 (−3.86, 0.06)
550	Low	10, 22	71.5 (7.7)	57.2 (5.2)	1 (0.00)	0.88 (0.09)	−0.83 (−1.96, 0.43)
579	Low	10, 20	25.5 (5.3)	33.8 (4.0)	1 (0.02)	1.47 (0.37)	3.10 (−0.80, 8.61)
579.2	Low	20,25	52.3 (6.3)	47.8 (6.2)	1 (0.00)	0.97 (0.16)	−0.18 (−1.96, 2.12)
	Low ave	11, 21.9	51.2 (6.2)	39.2 (5.4)	1	0.72 (0.27)	−1.44 (0.92)
522	High	10, 24	146.7 (10.8)	134.5 (10.7)	1 (0.01)	0.79 (0.10)	−1.43 (−2.68, 0.03)
537	High	10, 24	148.8 (6.9)	135.4 (7.2)	1 (0.00)	0.81 (0.07)	−1.33 (−2.19, −0.33)
566	High	10,22	123.5 (8.7)	110.6 (8.1)	1 (0.00)	0.93 (0.11)	−0.46 (−1.75, 1.13)
583	High	10, 24	123.0 (7.0)	116.9 (8.1)	1 (0.00)	0.99 (0.09)	−0.08 (−1.10, 1.12)
587	High	10, 24	142.4 (8.5)	129.1 (10.4)	1 (0.00)	0.85 (0.10)	−1.00 (−2.28, 0.28)
	High ave	10, 23.6	136.9 (5.7)	125.3 (4.9)	1	0.87 (0.04)	−0.86 (0.26)
−	G0 ancestor	15, −	148.5 (6.6)	−	1 (0.00)	−	−

Column headings are: 1° Line, first-order MA line; 1° Fitness, fitness group (high or low) of the 1° MA line; n_sublines, number of sublines (1° control pseudolines, 2° MA) within a 1° MA line; *W_0_*, absolute fitness of the 1° controls; *W_MA_*, absolute fitness of the 2° MA lines; *w*_0_, relative fitness of the 1° controls (defined = 1); *w_MA_*, relative fitness of the 2° MA lines; Δ*M_w_*, per-generation % change in *w*. Line 579.2 is the re-assay of line 579 (see text). Shaded rows are mean values of High and Low 1° fitness groups; the contribution of line 579 is the unweighted mean of the two assays. “G0 ancestor” is the common ancestor of the 1° MA lines in this assay. SEMs in parentheses except Δ*M_w_*, 95% CI in parentheses; see *Materials and Methods* for details of calculations.

#### Data analysis:

Absolute fitness (*W*) is defined as the lifetime reproductive output of an individual worm. We calculated a demographic measure of relative fitness (*w*) by the method of Charlesworth (1994), following Peters *et al.* (2003). $w=\sum_{x}e^{-r_{0}x}l_{x}m_{x}$, where $l_{x}m_{x}$ is the product of survivorship to and fecundity at day *x* and *r_0_* is the mean intrinsic rate of increase of control (1° MA) pseudolines, calculated by solving the equation ${\overset{–}{w}}_{0}=\sum_{x}e^{-r_{0}x}\overset{–}{l_{x}m_{x}}=1$, using the average *l_x_* and *m_x_* values of all control lines; *x* = 4.75, 5.75, and 6.75 for the first, second, and third day’s reproduction (Vassilieva and Lynch 1999). This calculation requires only one estimate of *r* per assay; because *r* is never calculated for individual worms, *w* is defined equal to 0 for individuals that did not reproduce. This measure accounts for differences in timing of reproduction as well as differences in the number of offspring and survivorship and is a more sensitive measure of fitness than is *W*.

##### Per-generation change in the mean (ΔM):

The change in mean phenotype (relative fitness, *w*) due to the cumulative effects of mutation is the product of genomic mutation rate (*U*) and the average effect of a mutation on the trait of interest E[*a*] [(Lynch and Walsh 1998) Ch. 12]. Fitness is scaled relative to the ancestral (1° MA) control mean, which is defined to equal 1. The difference between the MA mean and the control mean, scaled as a fraction of the control mean, $\frac{{\overset{–}{w}}_{MA}-{\overset{–}{w}}_{0}}{{\overset{–}{w}}_{0}}=\frac{{\overset{–}{w}}_{MA}-1}{1}$, so the per-generation rate of change of mean fitness Δ*M_w_* =$\frac{{\overset{–}{w}}_{MA}-1}{t}$, where *t* (defined as Gmax) is the number of generations of MA. An individual *i* is assigned a per-generation deviation in relative fitness Δ*M_w,i_ = *$\frac{w_{i}-1}{t_{i}}$. Because the control mean is the same in all groups $\left({\overset{–}{w}}_{0}\;=\;\;1\right)$, this quantity can be meaningfully compared between groups in which the trait mean differs and is straightforwardly quantified as slope of the regression of *w* on Gmax. Here and throughout we designate properties of the ancestral 1° control with a subscripted *0* and the same property in the 2° *MA* stocks with a subscripted *MA* (*e.g.*, *W_0_ vs. W_MA_*)

We compared Δ*M_w_* between fitness groups using restricted maximum likelihood as implemented in the MIXED procedure of SAS v. 9.21. The relevant independent variables are *Gmax* (generations of MA, 150 for 2° MA lines, and 0 for the 1° control); *Treatment* (a categorical variable equivalent to *Gmax*, 1° control *vs.* 2° MA), starting *Fitness* (high or low fitness of the 1° MA lines), *Line* (1° MA line, nested within *Fitness*), *Subline* (2° MA subline, nested within *Line*), and *Replicate* (nested within *Subline*). Gmax (=Treatment) and Fitness are fixed effects; the other effects are random. Degrees of freedom were determined by the Kenward-Rogers method. Significance of random effects was assessed by likelihood-ratio test (LRT) in which the likelihoods of the models with and without the term(s) of interest are compared; twice the difference between the log-likelihoods of the two models is expected to be χ^2^ distributed with degrees of freedom equal to the difference in the number of parameters estimated in the two models.

We assessed the model *w = Gmax + Gmax x Fitness + Line*(*Fitness × Treatment*) *+ Subline*[*Line*(*Fitness × Treatment*)] *+ Replicate*{*Subline*[*Line*(*Fitness ×Treatment*])}; the among-replicate variance is the residual variance. Note that defining mean relative fitness (*w*) of each ancestral 1° MA line equal to 1 is equivalent to (1) constraining the main effect of fitness to equal zero and (2) constraining the among-Line variance of 1° controls to equal zero. Therefore, the main effect of Fitness is not included in the model. The among-group components of variance for each random effect (1° line, subline, and replicate) were estimated separately for each Fitness × Treatment combination. A significant effect of Gmax in the above analysis indicates that Δ*M_w_* differs from zero; a significant Gmax × Fitness interaction indicates that Δ*M_w_* differs between the two fitness groups. SAS code is shown in Table S1.

##### Per-generation change in the among-line variance (V_M_):

In an MA experiment, one-half of the difference in the among-line component of variance between the MA lines (*V_L,MA_*) and the ancestral control pseudolines (*V_L,0_*) is the genetic variance resulting from new mutations; dividing by the number of generations of MA (*t*) yields the per-generation mutational variance, *V_M_* = $\frac{V_{L,MA}-V_{L,0}}{2t}$, where *V_M_* is equal to the product of the genomic mutation rate, *U*, and the average squared mutational effect, E[*a*]^2^ [(Lynch and Walsh 1998) Ch. 12]. Here, *V_M_* is represented by (half) the variance among 2° sublines within each 1° line. Comparisons of variances between groups are only meaningful if the means are equal; because mean fitness decreases with MA, relative fitness (*w*) of 1° control and 2° MA individuals is scaled to the mean of an individual’s own group, *i.e.*, control data are divided by the control mean (= 1) and MA data are divided by the mean of the 2° MA worms within each 1° line, so $w_{0}^{*}=\frac{w_{i}}{{\overset{–}{w}}_{0}}$ and $w_{MA}^{*}=\frac{w_{i}}{{\overset{–}{w}}_{MA}}$. The variance of the rescaled data, Var(w*), is the variance of the raw values divided by the square of the mean, the “opportunity for selection” (Crow 1958) and is the most appropriate measure of variance for a trait under directional selection (Houle 1992; Wade 2006). Scaling the variance in *w* by the MA mean rather than the ancestral mean is appropriate if mutational effects are multiplicative. We refer to Var(w*)/2*t* as V_X*_ where the *X* refers to the relevant variance component.

We first investigated whether the among-pseudoline variance in any 1° control differed significantly from zero by comparing the likelihood of the model w* = *Subline* + *Replicate*(*Subline*) for each 1° ancestral control against the model w* = *Replicate*(*Subline*). In only one 1° line is the *P* value of the LRT less than 0.4 (line 522, 0.14 < *P* < 0.15), so we pooled the ancestral control data over 1° lines to calculate the average among-pseudoline variance of the 1° controls and compared the likelihood of the model with the among-pseudoline variance included against the model with the among-pseudoline variance constrained to zero. The restricted maximum likelihood estimate of the among-pseudoline variance in the ancestral 1° controls is 0, (LRT, 1 df, χ^2^ = 0.0, *P* ≈ 1), so the 1° ancestral controls are ignored in analyses of V_M*_ unless noted otherwise.

We tested the hypothesis that V_M*_ differs between high-fitness and low-fitness 1° lines by comparing a model in which the among-subline variance was estimated separately for high-fitness and low-fitness 1° lines against a model with a single among-sub-line variance; the among-replicate (residual) variance was estimated separately for each 1° MA line. SAS code is shown in Table S2.

##### Bateman-Mukai (B-M) estimates of the genomic deleterious mutation rate (U_MIN_) and the average effect of a new mutation (E[a]_MAX_):

The “Bateman-Mukai” method is widely used to estimate mutational parameters from MA data (Halligan and Keightley 2009). There are several important caveats (see next paragraph), but if it is assumed that all mutations have equal effects, 2(Δ*M*)^2^/*V_M_* provides a downwardly biased estimate of the genomic (diploid) mutation rate for alleles that affect the trait (here relative fitness, *w*), *U_MIN_*, and *V_M_*/(2Δ*M*) provides an upwardly-biased estimate of the average effect of a mutation on the trait, E[*a*]*_MAX_* (Bateman 1959; Mukai 1964). The B-M estimators only yield consistent results (*i.e.*, Δ*M* = *U* × E[*a*] and *V_M_* = *U* × E[*a*]^2^) when *V_M_* is measured on the scale of the common ancestor of the MA lines. Estimates of Δ*M_w_* and *V_M_* were obtained for each 1° line by means of a bootstrap procedure (Baer *et al.* 2006). Data (MA and 1° controls) were resampled with replacement at the level of subline (*i.e.*, all replicates within a subline were included in each resample) and *w_0_* and *w_MA_* calculated from the unweighted line means of each resample. Variance components were estimated from each resample from the linear model *w* = *Subline* + *Replicate*(*Subline*) for 1° control and 2° MA lines separately; Δ*M_w_* = $\frac{{\overset{–}{w}}_{MA}-{\overset{–}{w}}_{0}}{{\overset{–}{w}}_{0}t}$ and *V_M_* = $\frac{V_{L,MA}-V_{L,0}}{2t}$ if *V_L,MA_-V_L,0_* > 0, else *V_M_* = 0. $\overset{–}{\Delta }{\overset{–}{M}}_{w}$ and ${\overset{–}{V}}_{M}$ were determined from the means of 1000 bootstrap replicates; approximate 95% confidence intervals were determined from the middle 95% of bootstrap replicates. *U_MIN_* and E[*a*]*_MAX_* were calculated from $\overset{–}{\Delta }{\overset{–}{M}}_{w}$ and ${\overset{–}{V}}_{M}$ via the B-M formulae given above.

Mutational parameters estimated by the B-M method must be interpreted with caution, for several reasons. First, in addition to the unrealistic assumption of equal mutational effects, the sampling covariance between *U_MIN_* and E[*a*]*_MAX_* is negative, although that consideration holds for all methods of inferring mutational properties from phenotypic data (*e.g.* maximum likelihood, see Begin and Schoen 2006). Second, certain outcomes can lead to nonsensical results (*e.g.* an “average mutational effect on relative fitness” >1 if *V_M_* is large and Δ*M* is small), although again that problem is shared by all methods of indirect inference. Third, and perhaps most importantly, the conceptual distinction between *U* and E[*a*] is inherently confounded [discussed in Baer *et al.* (2007)]. Nevertheless, B-M estimates are largely sensible on a broad scale (*e.g. U_MIN_* is invariably greater in flies than in fungi).

## Results

### Per-generation change in mean relative fitness (ΔM_w_)

**Figure 2:**
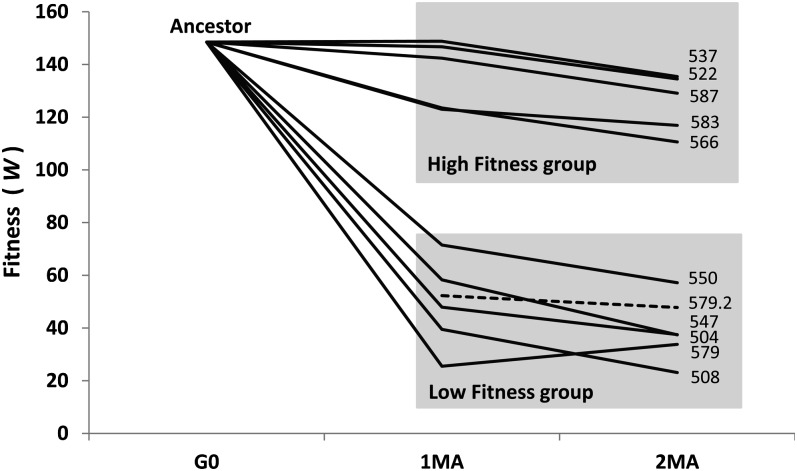
Evolutionary trajectories of absolute fitness (*W*) over 400 generations of MA. Values of absolute fitness are those reported in Table 1. Time points are 0 (G0), 250 generations (1MA), and 400 generations (2MA) of MA. The dashed line depicts the re-assay of Line 579 (see *Materials and Methods* for details).

The evolutionary trajectories of the ten 1° MA lines from generation 0 to 400 are depicted in Figure 2. Δ*M_w_* did not differ significantly between the high-fitness and low-fitness 1° MA lines (high-fitness Δ*M_w_* = −0.86 × 10^−3^/generation; low-fitness Δ*M_w_* = −1.44 × 10^−3^/generation, *P* > 0.18; Table 1). Averaged over all 10 1° lines, the mean Δ*M_w_* = −1.15 × 10^−3^/generation, which is remarkably close to the value of Δ*M_w_* calculated from the original set of N2 MA lines after 200 generations of MA [Δ*M_w_* = −1.19 × 10^−3^/generation, recalculated from data reported in Baer *et al.* (2005)].

### Mutational variance (V_M_)

Summary statistics of variances are presented in Table 2. V_M__*_ did not differ significantly between high-fitness and low-fitness groups. Averaged over all 101° lines, V_M__*_ was 2.94 × 10^−4^, again remarkably similar to the value of V_M*_ calculated from the original set of N2 MA lines after 200 generations of MA [V_M*_ = 3.19 × 10^−4^/generation, recalculated from data reported in Baer *et al.* (2005)].

**Table 2:** Summary statistics of variance in relative fitness

1° Line	1° Fitness	n_sublines (1°, MA)	V_L*,1°_	V _L*,MA_	V_M*_ (×10^4^)	V_E*,1°_	V_E*,MA_	$h_{M}^{2}$ (×10^3^)
504	Low	10, 22	0.02 (0.04)	0.17 (0.15)	5.25 (5.12)	0.64 (0.15)	0.94 (0.21)	0.73 (0.75)
508	Low	10, 21	0.02 (0.03)	0.01 (0.03)	0.22 (0.88)	0.59 (0.10)	1.18 (0.17)	0.28 (1.08)
547	Low	10, 22	0.08 (0.09)	0.16 (0.11)	3.61 (3.80)	0.80 (0.27)	1.06 (0.22)	0.40 (0.43)
550	Low	10, 22	0	0.02 (0.04)	0.82 (1.20)	0.66 (0.14)	0.79 (0.17)	0.13 (0.20)
579	Low	10, 20	0.12 (0.12)	0.01 (0.03)	0.15 (0.66)	1.67 (0.45)	1.26 (0.21)	0.01 (0.05)
579.2	Low	20,25	0.02 (0.04)	0.21 (0.13)	6.52 (4.60)	1.45 (0.24)	1.73 (0.25)	0.42 (0.29)
	Low ave	10, 21.4	0.04 (0.02)	0.09 (0.03)	2.65 (1.13)	0.85 (0.18)	1.09 (0.12)	0.35 (0.10)
522	High	10, 24	0.06 (0.04)	0.16 (0.06)	3.57 (2.40)	0.24 (0.04)	0.21 (0.04)	1.61 (1.08)
537	High	10, 24	0.00 (0.00)	0.07 (0.04)	2.21 (1.40)	0.19 (0.06)	0.27 (0.05)	0.98 (0.62)
566	High	10,22	0.02 (0.02)	0.09 (0.06)	2.33 (1.90)	0.28 (0.05)	0.35 (0.06)	0.73 (0.58)
583	High	10, 24	0.00 (0.00)	0.10 (0.04)	3.29 (1.30)	0.15 (0.04)	0.26 (0.04)	1.59 (0.62)
587	High	10, 24	0.00 (0.00)	0.14 (0.08)	4.76 (2.60)	0.28 (0.04)	0.39 (0.08)	1.41 (0.73)
	High ave	10, 23.6	0.02 (0.01)	0.11 (0.02)	3.23 (0.46)	0.23 (0.03)	0.30 (0.03)	1.26 (0.17)
—	All lines	10, 22.8	0.04 (0.02)	0.10 (0.02)	2.94 (0.50)	0.54 (0.13)	0.69 (0.15)	0.81 (0.18)

Column headings are: 1° Line, first-order MA line; 1° Fitness, fitness group (high or low) of the 1° MA line; n_sublines, number of sublines (1*°* control pseudolines, 2*°* MA) within a 1*°* MA line; V_L*,1°_,among-line variance of 1*°* control pseudolines; V_M*_, mutational variance; V_E*,1°_,environmental (within-line) variance of the 1*°* controls; V_E*,MA_, environmental (within-line) variance of the 2*°* MA sub-lines; $h_{M}^{2}$, mutational heritability (V_M*_/V_E*_); V_E*_ here is the average of the 1*°* controls and the 2*°* MA lines. Line 579.2 is the re-assay of line 579 (see text). Shaded rows are mean values of high and low 1*°* fitness groups; the contribution of line 579 is the unweighted mean of the two assays. SEMs in parentheses. All variances standardized by the relevant group mean; see *Materials and Methods* for details of calculations.

One extremely consistent feature of MA experiments is that the environmental component of variance (*V_E_*) increases with MA (Baer 2008b), and this study is no exception. V_E*_ increased by approximately 20% over 150 generations in both high-fitness and low-fitness 1° lines, approximately 0.1%/generation. In the original experiment, V_E*_ increased on average approximately 5% over 200 generations of MA, but the increase was almost 4X greater in the low-fitness 1° lines than in the high-fitness 1° lines (Table 2). One consequence of the different *V_E_*s between the two fitness groups is that the mutational heritability (*V_M_/V_E_*) is about fourfold greater in the high-fitness 1° lines than in the low-fitness lines.

### Bateman-Mukai (B-M) mutation parameters U_MIN_ and E[a]_MAX_

B-M mutation parameters are presented in Table 3. Three features of the results are apparent: (1) estimates of E[*a*]*_MAX_* from lines in which Δ*M_w_* is very small (lines 579, 583) were nonsensical because the average effect of a mutation on relative fitness cannot be greater than 1; (2) the median estimates of *U_MIN_* were larger and median estimates of E[*a*]_MAX_ were smaller in the low-fitness lines than in the high-fitness lines; however, (3) averaged over all 10 lines, median values of *U_MIN_* (0.022) and E[*a*]*_MAX_* (−0.057) were again very similar to the average values measured after 200 generations of MA in the original experiment (*U_MIN_* = 0.018, E[*a*]*_MAX_* = −0.073).

**Table 3:** Summary statistics of Bateman-Mukai estimates of mutation parameters

1° Line	1° Fitness	Δ*M_w_* (×10^3^)	*V_M_* (×10^4^)	*U_MIN_*	E[*a*]_MAX_
504	Low	−2.84	1.84	0.09	−0.04
508	Low	−2.80	0.07	2.35	−0.00
547	Low	−2.22	1.10	0.09	−0.02
550	Low	−0.83	0.63	0.02	−0.04
579	Low	3.10	0.31	—	—
579.2	Low	−0.18	6.44	0.00	−1.84
	Low Mean/Median	−1.44 / -2.22	1.40 / 1.10	0.51 / 0.09	−0.39 / −0.03
522	High	−1.43	1.81	0.02	−0.06
537	High	−1.33	1.38	0.03	−0.05
566	High	−0.46	1.81	0.00	−0.20
583	High	−0.08	3.12	0.00	−1.91
587	High	−1.00	3.13	0.02	−0.16
	High Mean/Median	−0.86 / −1.00	2.25 / 1.81	0.01 / 0.01	−0.48 / −0.16
—	All lines Mean/Median		1.83 / 1.81	0.26 / 0.02	−0.43 / −0.06

Column headings are: 1° Line, first-order MA line; 1° Fitness, fitness group (high or low) of the 1° MA line; *V_M_*, mutational variance scaled by the 1° control mean; *U_MIN_*, Bateman-Mukai estimate of genomic mutation rate for fitness; E[*a*]_MAX_. Bateman-Mukai estimate of average effect of a new mutation on fitness. Line 579.2 is the re-assay of line 579 (see text). Shaded rows are mean/median values of High and Low 1° fitness groups. The contribution of line 579 is the unweighted mean of the two assays. See *Materials and Methods* for details of calculations.

## Discussion

This study was motivated by two sets of empirical findings. First, Ávila *et al.* (2006) performed an analogous second-order MA study with a single high-fitness first-order MA line of *Drosophila melanogaster* and reported that both Δ*M_w_* and *V_M_* increased, which they attributed to an increase in *U* in the second-order MA lines. Second, Agrawal and his colleagues have performed several experiments with stocks of *D. melanogaster* in which starting fitness was manipulated either environmentally (Agrawal and Wang 2008) or by means of one or two mutations of large effect (Sharp and Agrawal 2012) and have consistently found that Δ*M_w_* increases with decreasing starting fitness and that fitness-dependent mutation rate provides the best explanation for the results. In addition, we recently performed a MA experiment in which mutations were accumulated at high and low temperature in two species of Caenorhabditis for which the high-temperature treatment was differently stressful and found that both dinucleotide microsatellite mutation rate and *U_MIN_* showed the signature of stress-dependent mutation (Matsuba *et al.* 2012). Finally, several studies have shown that deleterious effects can be magnified under physiologically stressful conditions (*e.g.*, Kondrashov and Houle 1994; Szafraniec *et al.* 2001, but see Agrawal and Whitlock 2010). If the stress is imposed from within by deleterious mutations it implies the existence of negative (synergistic) epistasis.

In contrast, we find here that, on average, neither the mutational decay of fitness Δ*M_w_* nor the mutational variance V_M*_ differ between low-fitness and high-fitness starting genotypes (but see *Discussion*). Taken at face value, the results of this study suggest that, on average, the mutational process does not vary in a consistent way with starting fitness. Moreover, comparison of the results from 2° MA with the results from the initial 1° MA experiment leads to the conclusion that, on average, the mutational properties of a given starting genotype remain remarkably constant (or at least highly repeatable) over hundreds of generations.

We can imagine several possible, nonexclusive reasons for the discrepancy between the absence of a significant relationship between starting fitness and mutational properties in this experiment and the results of the experiments mentioned above. First, with respect to the second-order MA experiment of Ávila *et al.* (2006), had we chosen only (for example) line 504, the results would have been nearly identical to those of Ávila *et al.* (2006), whereas had we chosen only line 583, we might be reporting on the long-term cessation of MA (Mackay *et al.* 1995). Importantly, both our results and those of Ávila *et al.* (2006) depend on the comparison with a control. Our controls were cryopreserved and had essentially no opportunity to evolve, whereas the control populations in fly MA experiments necessarily have the opportunity to evolve. However, there is sometimes considerable assay-to-assay variability in the absolute fitness of the N2 strain of *C. elegans*, as evidenced by the twofold difference in the absolute fitness of the 1° control of line 579 [Table 1; also see Figure 2 of Vassilieva *et al.* (2000)]. In fact, the re-assay of line 579 is quite revealing, because although it seems very likely that the apparent increase in fitness observed in the first assay is an artifact due to a compromised control, the results of the second, larger assay reinforce the conclusion that line 579 does not decrease in fitness by very much.

In contrast to the unavoidable limitation of the Ávila *et al.* (2006) experiment inherent in having to generalize from a single starting MA genotype, the finding of Sharp and Agrawal (Sharp and Agrawal 2012) that mutation rate increases with decreasing starting fitness is rather amazingly robust: 9/9 MA stocks initiated from different low-fitness genotypes declined in fitness faster than wild-type [see Figure 1 in Sharp and Agrawal (2012)]. A distinct possibility is that there is some consistent difference between the mutational processes of worms and flies, either with respect to input of DNA damage or in some feature of the DNA repair mechanism (Denver *et al.* 2003). Wang and Agrawal (in press) have shown that flies in good and poor physiologic condition predominantly use different mechanisms to repair double-strand breaks (although the difference was in the opposite direction predicted by their condition-dependent mutation hypothesis).

Nevertheless, there are several hints of an underlying relationship between starting fitness and subsequent mutation rate. First, and most obviously, the point estimate of the mean Δ*M_w_* of the low-fitness lines is ~2/3 greater than that of the high-fitness lines, and the difference is approximately twofold if the medians are considered. Second, the three lines that decrease in fitness most rapidly are low-fitness lines (*P* = 0.0833, exact probability). Third, the median *U_MIN_* of the low-fitness lines is tenfold greater than that of the high-fitness lines (0.087 *vs.* 0.0065) with a concomitant fivefold reduction in the average mutational effect E[*a*]*_MAX_* (0.033 *vs.* 0.156).

These nonsignificant trends highlight two issues concerning the power of an experiment to detect a significant association between starting fitness (or any generic property of the organism) and subsequent MA. The first issue is obvious: there is a trade-off between the number of starting genotypes that can be included and the number of MA lines per genotype that can be included. In this experiment we included twice as many starting genotypes as we have ever assayed before, but with only half the MA lines and half as many ancestral pseudolines per genotype. Obviously a larger experiment would permit finer resolution of the mutational properties of each genotype, and recent technological developments (*i.e.*, large-particle flow cytometry, aka a “worm sorter”) now permit much larger-scale phenotyping than we were able to achieve counting worms by hand. The second issue is somewhat more subtle; even supposing we were able to characterize (say) Δ*M_w_* of each 1° line to sufficient precision to where every line could be discriminated from every other line, and further supposing the point estimates from this experiment are the true values, we would be left with a rank order of (say) Δ*M_w_* of LLLHHHLHLH, where L and H represent high- and low-fitness 1° lines. No evolutionary biologist will be surprised by a situation in which within-group variation outweighs among-group variation, and viewed in that light our results are not only not surprising but might have been expected.

One final observation deserves mention. During the first 250 generations of MA, the five low-fitness 1° MA lines declined in fitness at an average rate of −0.26%/generation and the five high-fitness lines declined at an average rate of only −0.03%/generation (calculated from the data in the fourth column of Table 1). Three of the five low-fitness lines maintained an average 2° Δ*M_w_* of −0.26%/generation, whereas no high-fitness line had an average 2° Δ*M_w_* of more than −0.15%/generation, or in other words, no high-fitness line evolved an average 2° absolute fitness (W) anywhere near as low as even the highest of the low-fitness lines (line 550, *W_MA_* = 57.2). That result suggests (1) three of the five low-fitness 1° lines are at least weak mutators, and (2) that high-fitness genotypes are at least “not very likely” (0/5) to evolve to be mutators of that magnitude.

## Conclusions

Fitness data from MA experiments typically are quite noisy, and this one is no exception. We draw three general conclusions from the results, in decreasing strength of confidence. First, and apparently quite robust, is that the basic mutational properties of the N2 strain of *C. elegans*, under these particular conditions, on average, remain very constant for hundreds of generations of MA. Second, different lines derived from a common ancestor may evolve quite different average mutational properties quite rapidly, even absent any boost from positive selection. Third, circumstantial evidence suggests that low-fitness genotypes evolve increased mutation rates more often than do high-fitness genotypes; there is no evidence that supports the opposite conclusion.

## Supplementary Material

Supporting Information
