# Supplementary material for: Invariance (?) of Mutational Parameters for Relative Fitness Over 400 Generations of Mutation Accumulation in Caenorhabditis elegans
Source: G3 (Bethesda). 2012 Dec 1;2(12):1497–503. doi: 10.1534/g3.112.003947 (PMC3516472; doi:10.1534/g3.112.003947)
Supplement: Supporting Information [file supp_2_12_1497__index.html]

Supporting Information 

# Invariance (?) of Mutational Parameters for Relative Fitness Over 400 Generations of Mutation Accumulation in *Caenorhabditis elegans*

## Supporting Information for Matsuba *et al.*, 2012

**Files in this Data Supplement:**

- Supporting Information - File S1 and Tables S1-S3 (PDF, 118 KB)
- Table S1 - SAS code for the "full model" (PDF, 92 KB)
- Table S2 - SAS code to compare among-2° sub-line variances between high-fitness and low-fitness 1° lines (PDF, 98 KB)
- Table S3 - "Going to backup", approximate effective population size (*Ne*) and the demarcation of Effective Neutrality (PDF, 73 KB)
- File S1 - Raw fitness data (.xlsx, 92 KB)
