# Supplementary material for: Invariance (?) of Mutational Parameters for Relative Fitness Over 400 Generations of Mutation Accumulation in Caenorhabditis elegans
Source: G3 (Bethesda). 2012 Dec 1;2(12):1497–503. doi: 10.1534/g3.112.003947 (PMC3516472; doi:10.1534/g3.112.003947)
Supplement: Supporting Information [file supp_2.12.1497_003947SI.pdf]

File S1
Raw Fitness Data

Available for download at http://www.g3journal.org/lookup/suppl/doi:10.1534/g3.112.003947/-/DC1.

Table S1

A)

```
PROC MIXED COVTEST DATA=<all data except line 579.1 only, not line 579>;
      CLASS Fitness Treatment Line Subline Replicate;
      MODEL w=Gmax Gmax*Fitness/DDFM=Kenwardroger;
      RANDOM Line/GROUP=Fitness*Treatment;
      RANDOM Subline(Line)/Group=Fitness*Treatment;
      REPEATED Replicate(Line Subline)/GROUP=Fitness*Treatment;
RUN;
```

In these analyses we included only the data for the re-assay of line 579 (i.e., "line 579.1"); doing so makes the comparison of the means of the different fitness groups more liberal and the estimate of the among-line variances more conservative; see Tables 1 and 2 in the main text. Variable names are: Fitness (1° High fitness vs. Low fitness), Treatment (G250 ancestral control vs. G400 MA), Line (1° MA line), Subline (2° MA line), Replicate (individual), Gmax (G250 vs. G400 MA generation); note that "Treatment" and "Gmax" are the same variable represented categorically and continuously, respectively. A) SAS code for the "full model".

Table S2   SAS code to compare among-2° sub-line variances between high-fitness and low-fitness 1° lines.

```
PROC MIXED COVTEST DATA=<MA lines only, line 579.1 not line 579>;
       CLASS Fitness Line Subline Replicate;
       MODEL w*=/DDFM=Kenwardroger;
       RANDOM Subline(Line)/Group=Fitness;
       REPEATED Replicate(Subline)/GROUP=Line;
RUN;

vs.

PROC MIXED COVTEST DATA=< MA lines only, line 579.1 not line 579>;
       CLASS Fitness Treatment Line Subline Replicate;
       MODEL w*=/DDFM=Kenwardroger;
       RANDOM Subline(Line);
       REPEATED Replicate(Subline)/GROUP=Line;
RUN;
```

In the code at top, the among-subline variance is estimated separately for each fitness group (RANDOM Subline/GROUP=Fitness); in the code at bottom a single among-sub-line variance is estimated. Note that the dependent variable is *w**, relative fitness standardized to the mean of the 1° line.

Table S3 "Going to backup", approximate effective population size (N_e) and the demarcation of Effective Neutrality.

1° Line	*1° Fitness*	W_0	*N backups (SE)*	N_e	$1/4N_e$
504	Low	58.3	21.0 (1.7)	1.16	0.22
508	Low	39.5	19.6 (1.5)	1.15	0.22
547	Low	47.9	11.9 (0.8)	1.08	0.23
550	Low	71.5	10.8 (1.0)	1.08	0.23
579	Low	52.3	15.1 (0.8)	1.11	0.23
	Low Mean		15.7 (0.4)	1.12	0.22
522	High	146.7	5.3 (1.0)	1.04	0.24
537	High	148.8	4.0 (0.6)	1.03	0.24
566	High	123.5	8.0 (1.0)	1.06	0.24
583	High	123.0	4.8 (0.4)	1.03	0.24
587	High	142.4	6.7 (0.8)	1.05	0.24
	High Mean		5.6 (0.5)	1.04	0.24

1° Line and *1° Fitness* are defined in the text; W_0 is mean absolute fitness of the 1° ancestor (from Table 1), *N backups* is the average number of times we "went to backup" for a 2° subline in that 1° line, N_e is the harmonic mean census size of a 1° line, where backup generations were assigned a census size equivalent to W_0 and bottleneck generations were assigned a census size of 1. In a population that fluctuates in census size over time, N_e is equivalent to the harmonic mean census size. The parameter of effective neutrality in a MA experiment is very close to $1/4N_e$ (Keightley and Caballero 1997), i.e., a mutation with a selection coefficient < $1/4N_e$ will be effectively neutral.

References

Keightley, P. D. and A. Caballero, 1997 Genomic mutation rates for lifetime reproductive output and lifespan in *Caenorhabditis elegans*. Proceedings of the National Academy of Sciences, USA 94: 3823–3827.
