# Supplementary material for: Invariance (?) of Mutational Parameters for Relative Fitness Over 400 Generations of Mutation Accumulation in Caenorhabditis elegans
Source: G3 (Bethesda). 2012 Dec 1;2(12):1497–503. doi: 10.1534/g3.112.003947 (PMC3516472; doi:10.1534/g3.112.003947)
Supplement: Supporting Information [file supp_2.12.1497_TableS1.pdf]

Table S1

A)

```
PROC MIXED COVTEST DATA=<all data except line 579.1 only, not line 579>;
      CLASS Fitness Treatment Line Subline Replicate;
      MODEL w=Gmax Gmax*Fitness/DDFM=Kenwardroger;
      RANDOM Line/GROUP=Fitness*Treatment;
      RANDOM Subline(Line)/Group=Fitness*Treatment;
      REPEATED Replicate(Line Subline)/GROUP=Fitness*Treatment;
RUN;
```

In these analyses we included only the data for the re-assay of line 579 (i.e., "line 579.1"); doing so makes the comparison of the means of the different fitness groups more liberal and the estimate of the among-line variances more conservative; see Tables 1 and 2 in the main text. Variable names are: Fitness (1° High fitness vs. Low fitness), Treatment (G250 ancestral control vs. G400 MA), Line (1° MA line), Subline (2° MA line), Replicate (individual), Gmax (G250 vs. G400 MA generation); note that "Treatment" and "Gmax" are the same variable represented categorically and continuously, respectively. A) SAS code for the "full model".
