# Supplementary material for: Invariance (?) of Mutational Parameters for Relative Fitness Over 400 Generations of Mutation Accumulation in Caenorhabditis elegans
Source: G3 (Bethesda). 2012 Dec 1;2(12):1497–503. doi: 10.1534/g3.112.003947 (PMC3516472; doi:10.1534/g3.112.003947)
Supplement: Supporting Information [file supp_2.12.1497_TableS2.pdf]

Table S2 SAS code to compare among-2° sub-line variances between high-fitness and low-fitness 1° lines.

```
PROC MIXED COVTEST DATA=<MA lines only, line 579.1 not line 579>;
      CLASS Fitness Line Subline Replicate;
      MODEL w*=/DDFM=Kenwardroger;
      RANDOM Subline(Line)/Group=Fitness;
      REPEATED Replicate(Subline)/GROUP=Line;
RUN;

vs.

PROC MIXED COVTEST DATA=< MA lines only, line 579.1 not line 579>;
      CLASS Fitness Treatment Line Subline Replicate;
      MODEL w*=/DDFM=Kenwardroger;
      RANDOM Subline(Line);
      REPEATED Replicate(Subline)/GROUP=Line;
RUN;
```

In the code at top, the among-subline variance is estimated separately for each fitness group (RANDOM Subline/GROUP=Fitness); in the code at bottom a single among-sub-line variance is estimated. Note that the dependent variable is *w**, relative fitness standardized to the mean of the 1° line.
