# Supplementary material for: Invariance (?) of Mutational Parameters for Relative Fitness Over 400 Generations of Mutation Accumulation in Caenorhabditis elegans
Source: G3 (Bethesda). 2012 Dec 1;2(12):1497–503. doi: 10.1534/g3.112.003947 (PMC3516472; doi:10.1534/g3.112.003947)
Supplement: Supporting Information [file supp_2.12.1497_TableS3.pdf]

Table S3 "Going to backup", approximate effective population size (N_e) and the demarcation of Effective Neutrality.

1° Line	*1° Fitness*	W_0	*N backups (SE)*	N_e	$1/4N_e$
504	Low	58.3	21.0 (1.7)	1.16	0.22
508	Low	39.5	19.6 (1.5)	1.15	0.22
547	Low	47.9	11.9 (0.8)	1.08	0.23
550	Low	71.5	10.8 (1.0)	1.08	0.23
579	Low	52.3	15.1 (0.8)	1.11	0.23
	Low Mean		15.7 (0.4)	1.12	0.22
522	High	146.7	5.3 (1.0)	1.04	0.24
537	High	148.8	4.0 (0.6)	1.03	0.24
566	High	123.5	8.0 (1.0)	1.06	0.24
583	High	123.0	4.8 (0.4)	1.03	0.24
587	High	142.4	6.7 (0.8)	1.05	0.24
	High Mean		5.6 (0.5)	1.04	0.24

1° Line and *1° Fitness* are defined in the text; W_0 is mean absolute fitness of the 1° ancestor (from Table 1), *N backups* is the average number of times we "went to backup" for a 2° subline in that 1° line, N_e is the harmonic mean census size of a 1° line, where backup generations were assigned a census size equivalent to W_0 and bottleneck generations were assigned a census size of 1. In a population that fluctuates in census size over time, N_e is equivalent to the harmonic mean census size. The parameter of effective neutrality in a MA experiment is very close to $1/4N_e$ (Keightley and Caballero 1997), i.e., a mutation with a selection coefficient < $1/4N_e$ will be effectively neutral.

<u>References</u>

Keightley, P. D. and A. Caballero, 1997 Genomic mutation rates for lifetime reproductive output and lifespan in *Caenorhabditis elegans*. Proceedings of the National Academy of Sciences, USA 94: 3823–3827.
